# Let it glow: genetically encoded fluorescent reporters in *Plasmodium*

**DOI:** 10.1186/s12936-024-04936-9

**Published:** 2024-04-20

**Authors:** Pia J. Thiele, Raquel Mela-Lopez, Stéphanie A. Blandin, Dennis Klug

**Affiliations:** 1 Inserm, CNRS, Université de Strasbourg, UPR9022/U1257, Mosquito Immune Responses (MIR), IBMC, F-67000 Strasbourg, France; 2 Institute of Physiology and Pathophysiology, Department of Molecular Cell Physiology, Philipps University Marburg, 35037 Marburg, Germany

**Keywords:** Malaria, *Plasmodium*, Fluorescent protein, GFP, Apicomplexa, Parasite

## Abstract

The use of fluorescent proteins (FPs) in *Plasmodium* parasites has been key to understand the biology of this obligate intracellular protozoon. FPs like the green fluorescent protein (GFP) enabled to explore protein localization, promoter activity as well as dynamic processes like protein export and endocytosis. Furthermore, FP biosensors have provided detailed information on physiological parameters at the subcellular level, and fluorescent reporter lines greatly extended the malariology toolbox. Still, in order to achieve optimal results, it is crucial to know exactly the properties of the FP of choice and the genetic scenario in which it will be used. This review highlights advantages and disadvantages of available landing sites and promoters that have been successfully applied for the ectopic expression of FPs in *Plasmodium berghei* and *Plasmodium falciparum*. Furthermore, the properties of newly developed FPs beyond DsRed and EGFP, in the visualization of cells and cellular structures as well as in the sensing of small molecules are discussed.

**Graphical Abstract:**

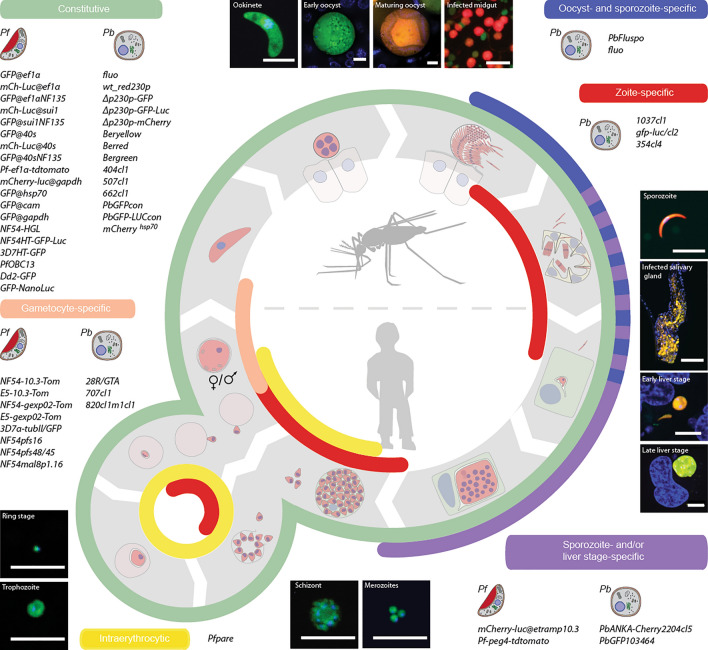

## Fluorescent proteins driving *Plasmodium* discoveries

**Fig. 1:**
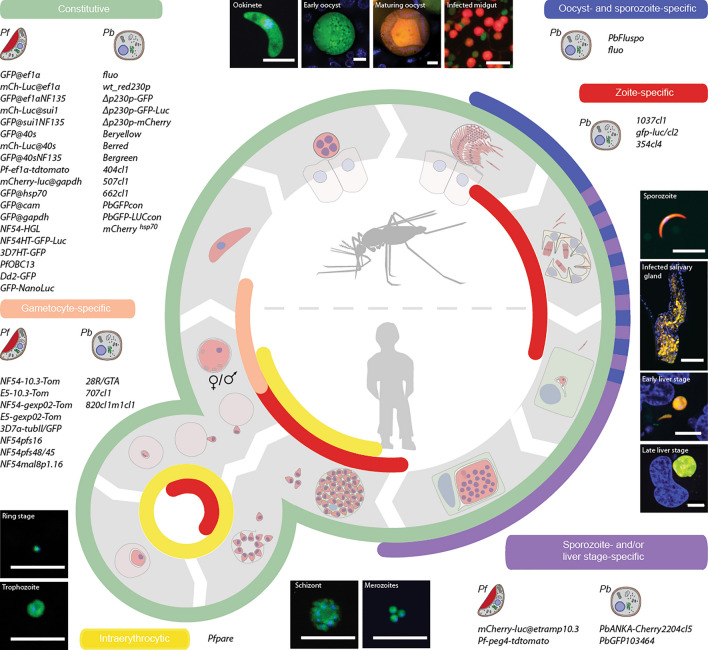
Stage-specific and constitutive expression of FPs along the *Plasmodium* life cycle. Human and mosquito outlines displayed in the center illustrate the division of the parasite cycle in insect-specific and vertebrate-specific stages. Fluorescent *P. berghei* and *P. falciparum* reporter lines driving expression of the respective FP from classical genomic loci used for ectopic gene expression are shown. Reporter lines are classified in constitutive, oocyst- and sporozoite-specific, zoite-specific, sporozoite- and/or liver stage-specific as well as gametocyte-specific. The colour code highlighting the different expression groups matches with that of the life cycle sections the respective lines express the fluorescent reporter. Images of the *P. berghei* fluo line are shown as an example of a reporter line with both constitutive (GFP driven by the *ef1a* promoter) and stage-specific FP expression (mCherry driven by the *CSP* promoter in late oocysts, sporozoites and early liver stages). Merged images showing GFP and mCherry expression in combination with nuclear staining are shown for all stages. The scale bar for all images is 10 µm except for midgut and salivary gland where it represents 100 µm. Please note that *P. falciparum* reporter lines making use of the *etramp10.3./peg4* promoter also display FP expression in gametocytes, although not shown here. Also, the *P. berghei* reporter line *PbGFP103464* is fluorescent in late liver stages only, while the reporter line *PbANKA-Cherry 2204cl5* is fluorescent in sporozoites and liver stages

*Plasmodium* parasites, the causative agents of malaria, have evolved a unique parasitic life style, which requires switching between a vertebrate and an insect host, oscillating from motile to immotile forms, as well as between protist-like, sessile, intracellular and facultative intracellular stages (Fig. 1). Each of these stages is equipped with its own set of proteins, many of them functioning at the host-parasite interface, being either secreted towards the plasma membrane to ensure motility and recognition of tissues or scavenging nutrients and remodeling host cells. Imaging of different *Plasmodium* stages on different scales has significantly contributed to a better understanding of the function of individual proteins and of parasite-specific structures (reviewed in detail in [1]). To gain insights into the biology of *Plasmodium* and other organisms, genetically encoded fluorescent proteins (FPs) have made a historical contribution as they allow the localization of compartments and proteins in vivo and in vitro thus also the visualization of dynamic processes. Although FPs fused to proteins can cause mis-trafficking or impair protein function, the possibility to visualize processes in living cells gives FPs decisive advantages over immunofluorescence-based methods, which require fixation and subsequent staining.

An extremely important dynamic process with significant implications for malaria pathology is the uptake of haemoglobin by the parasite when developing within a red blood cell (RBC). While the parasite grows within human RBCs, it develops from a ring stage into a trophozoite that subsequently matures into a schizont. The latter divides into a number of merozoites that upon release, invade new RBCs and continue asexual replication. During its development inside RBCs, *Plasmodium* main source of amino acids is haemoglobin. However, the parasite is separated from the RBC cytoplasm by a parasitophorous vacuole membrane impeding haemoglobin uptake. A recent study shed light on the pathway that initiates endocytosis of haemoglobin by using FPs to tag the Kelch propeller protein K13, a protein involved in artemisinin resistance, and its interactome [2]. It also demonstrated that reduced expression of K13 can slow down endocytosis, making anti-malarials, such as artemisinin, less effective [2]. While the majority of merozoites that invade RBCs replicate asexually, a minority commits to become gametocytes, the only form that is infectious to mosquitoes. How sexual commitment is regulated and how individual parasites decide their developmental path remained elusive for a long time. The protein gametocyte development 1 (GDV1) was recently discovered to be a key regulator in this process. By fusing GDV1 with a reporter consisting of GFP and a destabilization domain, gametocyte conversion was shown to be highly dependent on the expression level of GDV1, which acts as a transcriptional activator of silenced gametocyte specific genes [3].

Mature male and female gametocytes, when ingested by a female *Anopheles* mosquito during a blood meal, become activated and subsequently fuse to form a zygote. The zygote develops into a motile stage, called ookinete, which crosses the midgut epithelium. Once the ookinete reaches the basal lamina, it transforms into a sessile oocyst which, after growing for up to two weeks, divides into thousands of motile sporozoites. During all these stage transitions, gametocyte to gamete(s), zygote to ookinete and oocyst to sporozoite(s), chromosomal rearrangements play an important role in DNA replication and distribution. Recently, the C-terminal fusion of kinsesin-8X to GFP enabled the representation of mitotic and meiotic parasitic stages in vivo with unprecedented accuracy [4]. Similarly, the production of interdomain GFP fusion proteins with the circumsporozoite protein, an important vaccine candidate for malaria prophylaxis, allowed new insights into the transport and processing of this important protein as well as into the formation of sporozoites during oocyst development [5]. Sporozoites emerging from oocysts, are released into the haemolymph and cross the salivary gland epithelium to reach the lumen from which they will be deposited with the saliva in the dermis of hosts upon blood feeding. Transmitted sporozoites actively move to enter the bloodstream, which transports them to the liver.

Using parasite lines expressing FPs at the sporozoite stage and software-based analysis to quantify movement patterns, it could be shown that sporozoites sense environmental elasticity to discriminate between different tissues [6] and that sporozoites of human and rodent infecting malaria species require no species-specific adaptations to traverse the skin of different hosts [7]. In the liver parasites invade hepatocytes and develop as exoerythrocytic forms (EEFs) until they undergo schizogony to form thousands of merozoites. In order to be efficiently released from infected hepatocytes, the parasites must disrupt the parasitophorous vacuole in a phospholipase-mediated process that subsequently leads to the death of the host cell and the detachment of merozoite-filled vesicles, the merosomes [8, 9]. Merozoites released from EEFs invade RBCs and complete the life cycle. As illustrated by these few examples here, the use of fluorescent proteins has made significant contributions to understand the life cycle of *Plasmodium* in general, and of stage-dependent adaptations and stage-to-stage transitions in particular. In addition to the classical use of genetically encoded FPs for labelling cells or subcellular structures by fusion with proteins of interest, a number of *Plasmodium* specific applications for FPs have been invented, which are discussed in more detail below.

## *Plasmodium*-specific applications of FPs

The unique life cycle of *Plasmodium* has led to the development of a number of applications where FPs have proven particularly useful. Transgenesis in *Plasmodium* requires usually subsequent purification of the transgenic parasites to ensure the absence of wild-type. This is usually done by creating isogenic populations founded by a single parasite. In the case of *Plasmodium falciparum*, isogenic lines can be produced by simply sowing individual parasites in wells and allowing them to grow in vitro. In contrast, the generation of isogenic populations in rodent malaria parasites requires the injection of single parasites into mice (~ 8–10 per transgene) to ultimately obtain multiple clones of transgenic parasites. Blood stage-specific expression of FPs enables fluorescence-activated cell sorting (FACS) of successfully transfected parasites immediately after transfection [10]. Besides saving time and animals, this method also preserves the genetic diversity of the strain as the parasite populations produced are homogenic to the transgene but polyclonally founded. A further improvement of the FACS-based approach is the fusion of FPs with luciferases. The expression of FPs enables the microscopic detection of parasites, but also the sorting with flow cytometry for different experimental set-ups. On the other hand, the luciferase fused to the FP makes it possible to visualize the sequestration of parasites in the vasculature and the spleen but also to compare the parasite burden in the liver in vivo (reviewed in detail in [11]). Other uses of parasites expressing FP-luciferase fusions are multiplexing approaches in vitro, e.g. for drug screening assays in *P. falciparum* [12] or the quantification of parasite burden in *Plasmodium berghei* [13]. Besides methodological facilitation, the use of FPs also contributed to better understand *Plasmodium* unique sexual reproduction. Dual fluorescent reporter lines with male and female gametocyte-specific FP expression allow monitoring of gamete fusion and ookinete conversion by flow cytometry. This evidenced how important the detachment of the erythrocyte membrane is for the subsequent fusion of male and female gametes [14]. Similarly, sexual hybridization of *Plasmodium* lines expressing differently coloured FPs can be exploited to bypass lethal gene defects in ookinetes and oocysts and allow the study of loss-of-function mutants in sporozoites and liver stages. Indeed, a loss-of-function parasite line constitutively expressing a fluorescent reporter can be crossed with a fluorescent reporter line expressing a FP of a different colour. When the gametes of both lines fuse, the functional allele of the fluorescent control rescues the lethal developmental defect of the loss-of-function transgene and allows the formation of an oocyst expressing both FPs. Subsequent cell division results in haploid sporozoites with different genotypes. A subset of them exclusively carries the loss-of-function allele that is trackable by its FP reporter. Cross-fertilization revealed that the prohibitin-like protein in *P. berghei* is not required for transmission and development of liver stages [15]. In contrast, cross-fertilized ferrochetalase-deficient *P. berghei* parasites are unable to complete liver development [16].

FPs have also been used to investigate transport processes in *Plasmodium*. Translocation of proteins across different membranes was studied by fusing GFP to murine dihydrofolate reductase (mDHFR). In the presence of membrane-permeable folate analogues (e.g. WR99210), the mDHFR adopts a very stable fold which prevents protein translocation across membranes. By adding the folate analogue, protein transport can be blocked in a time- and dose-dependent manner, which can be followed in real time by visualizing the attached GFP. Using this method, the export of parasite proteins into the cytoplasm of infected RBCs was shown to require protein unfolding [17] and, more recently, the same approach was used to jam protein import into the parasite apicoplast [18]. As these examples highlight, genetically encoded FPs are often fused to other proteins to extend their utility. In the following sections, we focus in particular on the monomeric expression of FPs to enable fluorescence-based detection of cells without approaching the subcellular level. Whilst this approach can be considered the simplest, the promoter driving FP expression and the landing site chosen can be critical to the progression of a project. Advantages and disadvantages of available promoters and landing sites as well as the properties of newly developed FPs for visualisation and small molecule sensing are discussed.

## Genomic loci used for ectopic gene expression (of FPs) in *P. berghei* and *P. falciparum*

Genetically encoded FPs can be integrated into the parasite genome in various ways. Common strategies include the labeling of endogenous proteins with FPs or the knockout of genes by replacing them with genetic cassettes encoding FPs. Of note, the insertion site might affect the expression level of the reporter, which can be a problem if fluorescence quantification is required to compare different mutants. In addition, it may not always be possible to integrate a FP, for example if a genetic cassette with a different genetic cargo is to be integrated or if only single bases within a coding sequence are to be edited. In this case it is often required to use a fluorescent line with ectopical expression of a suitable FP at a separate integration site. In order to save time, resources and, in the case of rodent-infecting *Plasmodium* species, animals for cultivation, already existing lines should be used when possible. Commonly used parasite lines expressing FPs either as a monomer or fused to luciferase are listed in Table 1 and 2 for *P. berghei* and *P. falciparum*, respectively. If a project requires the creation of a line with the expression of a specific FP or a specific expression pattern, it is often recommended to use one of the already utilized landing sites for ectopic transgenes. Historically, the first targeted locus to integrate ectopically expressed transgenes in *P. berghei* was the dihydrofolate reductase* (dhfr)* gene because the selection marker *Tg*DHFR-TS would restore the essential function of the endogenous gene while also providing resistance to the drugs pyrimethamine and WR99210 (see "List of abbreviations") [19]. Later studies in *P. berghei* focused on redundant genes like the C- and D-rRNA genes (C-/D-rRNA) [20–22] and the p230p gene, a member of the s48/45 domain containing 6-cysteine (6-cys) protein family [23]. In *P. falciparum* integrations into the locus of another 6-cys family member, *Pfs47*, do not interfere with life cycle progression [24], which made it a common landing site [25]. Still, *P. falciparum* lines lacking *Pfs47* expression are unable to infect native African malaria vectors. A possible alternative to the *Pfs47* locus could be the *lisp1* locus, which has recently been explored in *P. falciparum* as target site for the ectopic expression of transgenes [26]. While *lisp1* may be required for efficient merozoite exit from the liver stages, it has no effect on other stages of the life cycle [27]. Further alternatively explored loci in *P. falciparum* have been *obc13* and *pare*, both believed to be redundant for the *Plasmodium* life cycle. *Pare* encodes a prodrug activation and resistance esterase that is dispensable for the parasite. In addition, the loss of *pare* makes the parasites resistant to the drug MMV011438 enabling negative selection [28]. The use of the obc13 locus, enabling constitutive reporter gene expression without affecting life cycle progression, is also promising [29]. The knockout of OBC13 has been described to have no phenotype in gametocytes in vitro, while in vivo oocyst numbers decrease slightly. Another potentially interesting landing site in *P. falciparum* is RH3, a putative pseudogene being transcribed in schizonts but not translated, possibly due to frameshift mutations [30, 31]. To further limit a possible interference with endogenous gene expression, transgenes in *P. berghei* have been placed into transcriptionally silent loci on chromosome 6 (SIL6) [32] and chromosome 12 (named here SIL12) [5, 33]. Similarly, the 3'UTR of the *cg6* gene in *P. falciparum* was used to host transgenes [34]. Intuitively, intergenic sequences are preferable to landing sites in coding regions. However, changes in the transcription of neighbouring genes can occur, which are often subtle and not easy to detect. For example, SIL12 is located near the coding sequence of the mitochondrial protein MPODD, potentially limiting the usability of this landing site for studies on mitochondrial function [35].

**Table 1:** List of reporter lines in *P. berghei* with FP expression in an otherwise unaltered genetic background**.** Compilation of transgenic lines with ectopic expression of FPs either as monomer or in fusion with luciferase

Parasite line	Fluorescent stages	Promoter	FP reporter	Selectable marker	Target site	RMgmDB	References
*fluo*^*α*^	Complete life cycle oocysts, sporozoites	*eEF1α/csp*	GFP/mCherry	removed	SIL12	–	[38]
*wt_red*_*230p*_	Complete life cycle	*eEF1α*	mCherry	tgDHFR-TS	230p	1227	[89]
*Δp230p-GFP*^*α*^	Complete life cycle	*hsp70*	GFP	removed	230p	1026	[44]
*Δp230p-GFP-Luc*^*α*^	Complete life cycle	*eEF1α*	GFP-Luciferase	removed	230p	1027	[44]
*Δp230p-mCherry*^*α*^	Complete life cycle	*eEF1α*	mCherry	removed	230p	–	[44]
*Beryellow*	Complete life cycle	*hsp70*	YFP	hDHFR	SIL6	948	[43]
*Berred*	Complete life cycle	*hsp70*	mCherry	hDHFR	SIL6	949	[43]
*Bergreen*	Complete life cycle	*hsp70*	GFP	hDHFR	SIL6	947	[43]
*404cl1*^*β*^	Complete life cycle	*eEF1α*	GFP	FACS	C-rRNA	–	[36]
*507cl1*^*β*^	Complete life cycle	*eEF1α*	GFP	FACS	230p	7	[10]
662cl1*^β^	Complete life cycle	*eEF1α*	GFP-Luciferase	FACS	C-rRNA	–	[10]
*PbGFP-LUCcon*^*β*^	Complete life cycle	*eEF1α*	GFP-Luciferase	FACS	230p	29	[10]
PbGFPcon*	Complete life cycle	*eEF1α*	GFP	tgDHFR-TS	C-rRNA	5	[21]
*PbANKA-Cherry 2204cl5*^*α*^	Sporozoites, liver stages	*uis4*	mCherry	removed	230p	-	[51]
PbGFP103464*	Liver stages	*lisp2*	GFP	tgDHFR-TS	C-/D-rRNA	593	[22]
*1037cl1*^*β*^	Merozoites, sporozoites	*ama1*	GFP-Luciferase	FACS	230p	32	[48]
gfp-luc/cl2*	Merozoites, sporozoites	*ama1*	GFP-Luciferase	tgDHFR-TS	C-/D-rRNA	200	[48]
354cl4*	Merozoites, sporozoites	*ama1*	GFP-Luciferase	tgDHFR-TS	D-rRNA	30	[49]
*28R/GTA*^*α*^	Female gametocytes All stages except female gametocytes	*P28* *α-tubulin II*	mRuby mNG	removed	230p	164	[50]
707cl1*	Gametocytes	*mdv-1/peg3*	GFP	tgDHFR-TS	C-rRNA	251	[14]
*820cl1m1cl1*^*α*^	Male gametocytes Female gametocytes	*mg1* *ccp2*	GFP RFP	removed	230p	164	[14]
PbFluspo*^γ^	Oocysts, sporozoites	*csp*	GFP	pbDHFR-TS	5’ UTR of csp	77	[52]
*mCherry*_*Hsp70*_	Complete life cycle	*hsp70*	mCherry	removed	230p	-	[9]

^*^ Single crossover integration

α Selection marker was removed through negative selection

β Parasite lines were generated without chemical selection and cloned by fluorescence activated cell sorting (FACS)

γ NK65 genetic background (other lines were made in the genetic background ANKA)

**Table 2:** List of reporter lines in *P. falciparum* with FP expression in an otherwise unaltered genetic background

Parasite line	Fluorescent stages	Promoter	FP reporter	Selectable marker	Target site	Strain	References
*GFP@ef1α*	Complete life cycle	*eEF1α*	GFP	Removed	*Pfs47*	NF54	[25]
*mCh-Luc@ef1α*	Complete life cycle	*eEF1α*	mCherry-Luciferase	Removed	*Pfs47*	NF54	[25]
*GFP@ef1α*_*NF135*_	Complete lifecycle	*eEF1α*	GFP	Removed	*Pfs47*	NF135	[25]
*mCh-Luc@sui1*	Complete life cycle	*sui1*	mCherry-Luciferase	Removed	*Pfs47*	NF54	[25]
*GFP@sui1*_*NF135*_	Complete life cycle	*sui1*	GFP	Removed	*Pfs47*	NF135	[25]
*GFP@40 s*	Complete life cycle	*40S*	GFP	Removed	*Pfs47*	NF54	[25]
*mCh-Luc@40 s*	Complete life cycle	*40S*	mCherry-Luciferase	Removed	*Pfs47*	NF54	[25]
*GFP@40s*_*NF135*_	Complete life cycle	*40S*	GFP	Removed	*Pfs47*	NF135	[25]
Pf-ef1a-tdTomato*	Complete life cycle	*eEF1α*	tdTomato	BSD	*Pfs47*	NF54^Pfs47attB^	[41]
*mCherry-luc@gapdh*	Complete life cycle	*gapdh*	mCherry-Luciferase	Removed	*Pfs47*	NF54	[47]
*GFP@hsp70*	Complete life cycle	*hsp70*	GFP	Removed	*230p*	NF54	[46]
*GFP@cam*	Complete life cycle	*cam*	GFP	Removed	*230p*	NF54	[46]
*GFP@gapdh*	Complete life cycle	*gapdh*	GFP	Removed	*230p*	NF54	[46]
*NF54-HGL*	Complete life cycle	*hsp70*	GFP-Luciferase	Removed	*Pfs47*	NF54	[45]
NF54HT-GFP–luc*	Complete life cycle	*eEF1α*	GFP-Luciferase	hDHFR	*Pfs47*	NF54	[40]
3D7HT-GFP*	Complete life cycle	*eEF1α*	GFP	hDHFR	*Pfs47*	3D7	[39]
*mCherry-luc@etramp10.3*	Liver stages, gametocytes, sporozoites	*etramp10.3/peg4*	mCherry-Luciferase	Removed	*Pfs47*	NF54	[47]
Pf-peg4-tdTomato*	Liver stages, gametocytes, sporozoites	*etramp10.3/peg4*	tdTomato	BSD	*Pfs47*	NF54^Pfs47attB^	[41]
*NF54-10.3-Tom*^*α*^	Gametocytes	*etramp10.3/peg4*	tdTomato	hDHFR-yFCU	*lisp1*	NF54	[26]
*E5-10.3-Tom*^*α*^	Gametocytes	*etramp10.3/peg4*	tdTomato	hDHFR-yFCU	*lisp1*	E5	[26]
*NF54-gexp02-Tom*^*α*^	Gametocytes	*gexp02*	tdTomato	hDHFR-yFCU	*lisp1*	NF54	[26]
*E5-gexp02-Tom*^*α*^	Gametocytes	*gexp02*	tdTomato	hDHFR-yFCU	*lisp1*	E5	[26]
*3D7*^α−tubII/GFP^*	Gametocytes	*α-tubulin II*	GFP	BSD	*3’hrp2*	3D7	[90]
*NF54*^pfs16^*	Gametocytes	*Pfs16*	GFP-Luciferase	Removed	*cg6*	NF54^attB^	[34]
*NF54*^pfs48/45^*	Gametocytes	*Pfs48/45*	GFP-Luciferase	Removed	*cg6*	NF54^attB^	[34]
*NF54*^mal8p1.16^*	Gametocytes	*Mal8p1.16*	GFP-Luciferase	Removed	*cg6*	NF54^attB^	[34]
Dd2-GFP*	Complete life cycle	*hsp70*	GFP	hDHFR	*cg6*	Dd2	[91]
*Pfpare*	Intraerythrocytic cycle	*Pfpare*	mNG	BSD	*Pfpare*	Dd2	[28]
*PfOCB13-GFP*	Complete life cycle	*hsp70*	GFP	Free	*OBC13*	NF54	[29]
*GFP-NanoLuc*	Complete life cycle	*40S*	GFP-T2A-NanoLuc	Free	*Pfs47*	NF54	[12]

Compilation of transgenic lines with ectopic expression of FPs either as monomer or in fusion with luciferase

^*^ Single crossover integration

α Parasite line potentially has a phenotype in the liver stage delaying egress of merozoites

## Promoters used to drive constitutive and stage-specific expression of FPs in *P. berghei* and *P. falciparum*

*Plasmodium* lines with constitutive or stage-specific cytoplasmic expression of different FPs have greatly simplified quantification, sorting and visualization of parasites. However, the application possibilities of a reporter line always depend on the promoter that controls the expression of the respective FP. Since the stages and activities of the promoters of the same gene can differ significantly between different *Plasmodium* species, a large number of fluorescent reporter lines with different applications has been created. Most reporter lines have been generated in *P. falciparum* and *P. berghei*, causing malaria in humans and rodents, respectively. *P. berghei* has proven to be particularly useful to study insect stages and their transmission, which is difficult to reproduce with *P. falciparum*. Application-related fluorescent reporter lines with constitutive gene expression in both parasite species enabled the use of fluorescence-based methods along the life cycle. The first promoter used to engineer reporter lines with constitutive expression was the 5’UTR of elongation factor 1α (*ef1α*) that can drive FP expression in both *P. berghei* [10, 21, 36–38] and *P. falciparum* [25, 39–41]. While the toolbox of constitutive promoters in *P. berghei* for ectopic gene expression is limited to *ef1α* and heat shock protein 70 (*hsp70*) which drives significantly stronger gene expression compared to *ef1α* [42–46], additional promoters have been used to engineer constitutive fluorescent reporter lines in *P. falciparum*, notably glyceraldehyde-3-phosphate dehydrogenase *(gapdh),* calmodulin *(cam)* as well as the promoters of the two housekeeping genes translation initiation factor SUI1 *(sui1)* and 40s ribosomal protein S30 *(40s)* [25, 46, 47]. *Cam* is a weaker driver compared to *gapdh* [46], and *sui1* as well as *40s* provide stronger gene expression than *ef1α* in *P. falciparum* [25]. Still, thus far, all tested constitutive promoters in *P. falciparum* are significantly weaker than the *P. berghei hsp70* promoter, which impedes their usefulness for applications requiring strong fluorescence.

Stage-specific transcriptional elements have also been used to generate reporter lines. For example, the promoter of the apical membrane antigen 1 (*ama1*), which guides gene expression in schizonts, merozoites and sporozoites, has been used to generate transgenic *P. berghei* lines expressing GFP-luciferase fusion proteins. While the expression of GFP in these lines enabled the selection of transgenic parasites by FACS, their primary use was the luciferase-based localization of sequestering schizonts in infected mice [48, 49]. Equally important, *Plasmodium* sexual commitment has been a long-term interest of researchers because the timing of gametocyte development as well as the percentage of viable gametocytes are crucial for transmission. Moreover, gametocytes persist upon treatment with most common anti-malarial drugs, which allows parasites to continue to be transmitted to mosquitoes even during treatment. Since gametocyte development in *P. falciparum* takes place in different morphologically recognizable stages, it was relatively simple to find promoters that switch on the expression of FPs in relation to the developmental status. Promoter of the helical interspersed subtelomeric protein GEXP02 (*gexp02*), Pfs16 (*pfs16*) as well as the protein Pfs48/45 (*pfs48/45*) have been used in *P. falciparum* to generate fluorescent reporter lines very early on during sexual commitment. Interestingly a subset of schizonts expressed tdTomato in the *gexp02* driven line although they were reported negative for the early gametocyte marker Pfs16 by immunofluorescence stainings [26]. Similarly, although placed under the control of the *pfs16* promoter, FP expression was also detected in asexual blood stages [26], while a previously generated reporter line using the same promoter has been reported to display a high degree of gametocyte specificity [34]. Whether this is a natural occurring expression pattern of both *gexp02* and *pfs16* promoters, or technical artefacts due to the size of the selected 5’UTR fragments or to locus-specific effects linked to different genomic integration sites, remains to be investigated. Besides promoters that are active early in gametogenesis, the *mal8p1.16* promoter was used to engineer a reporter line with high expression levels in mature gametocytes [34].

In contrast to *P. falciparum,* different gametocyte stages are not recognizable in *P. berghei*. The generation of gametocyte reporter lines in *P. berghei* has, therefore, focused on sex-specific expression. The promoter of the male development gene 1 (*mdv-1/peg3*) has been shown to induce higher transcriptional activity in female than in male gametocytes and was used to generate a fluorescent reporter line enabling sex differentiation by flow cytometry [14]. Similarly, the surface protein p28 and the LCCL domain-containing protein CCP2 are expressed in female but absent in male gametocytes. The *ccp2* promoter has been used in combination with the male-specific promoter of the dynein heavy chain (*mg1*) to engineer a dual colour reporter line with male- and female-specific expression of the respective FPs [14]. Similarly, a *P. berghei* reporter line was developed using the *p28* promoter to express mRuby in combination with mNeonGreen-tagged (mNG) α-tubulin II [50]. This line expresses mNG-tagged α-tubulin II in all stages except female gametocytes, while female gametocytes specifically express mRuby. While originally used as driver for gene expression in mature gemetocytes, it became evident with time that the *etramp10.3/peg4* promoter also activates transcription in oocysts, sporozoites and liver stages (Fig. 1, Table 2). As a consequence the promoter experienced a revival for the design of fluorescent reporter lines to simplify the study of insect and pre-erythrocytic stages in *P. falciparum* [26, 41, 47]. The orthologue of *etramp10.3/peg4* in *P. berghei*, named upregulated in sporozoites 4 (*uis4*), was used to generate a reporter line driving the expression of mCherry to study sporozoite motility in vivo [51].

Of note, the promoter of the circumsporozoite protein (*csp*), driving very high expression levels especially in sporozoites, was used to engineer one of the first reporter lines in *P. berghei* [52]. More recently the *csp* promoter was combined with the *ef1α* promoter in the *fluo* reporter line to drive sporozoite-specific mCherry expression and constitutive GFP expression, respectively [38] (see also Fig. 1). The parasite burden in mosquitoes can be adjusted based on the determination of parasitaemia and gametocytaemia in infected mice by flow cytometry (GFP), while the strong mCherry expression in insect stages enables software-based analysis of sporozoite motility that was not possible when using *ef1α* driven reporter lines. Although some promoters presented above (e.g. *uis4* and *etramp10.3/peg4*) display transcriptional activity in liver stages, they are not exclusively expressed in this stage. In contrast the liver-specific protein 2 (*lisp2*) 5’UTR drives GFP expression only in *P. berghei* late liver stages [22]. Of note, all lines presented here display cytoplasmic localization of their respective expressed FP, and have been generated by fluorescence cassette insertion in classical genomic loci used for ectopic gene expression (*Pb*: 230p and c-/d-rrna; *Pf*: 230p, Pfs47, cg6 and lisp1) or in intergenic loci (*Pb*: SIL6, SIL12 and 5’UTR of CSP; *Pf*: 3’UTR of cg6). They can thus be used as standards for fluorescence based applications. All discussed *P. falciparum* and *P. berghei* reporter lines are listed in Table 1 and Table 2, respectively, according to their expression profile as in Fig. 1. Transgenic *P. berghei*, *Plasmodium yoelii* or *Plasmodium chabaudi* lines with different genetic modifications or reporter cassettes in non-classical genomic loci can be found in the rodent malaria database [53].

## FPs as sensors

**Fig. 2:**
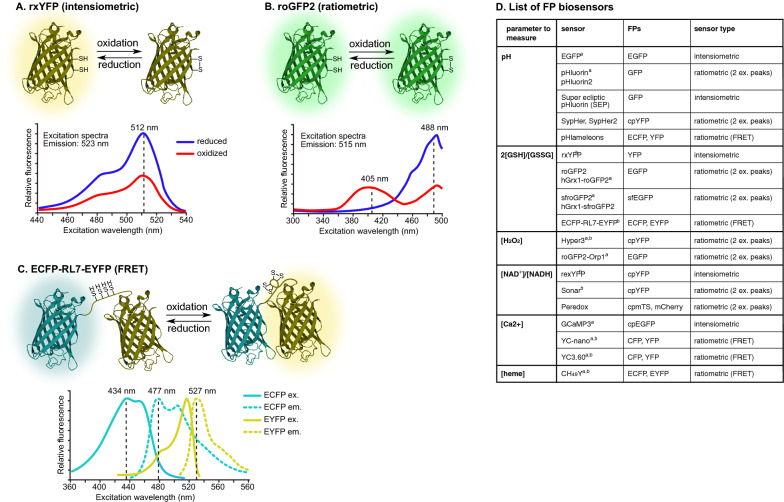
Fluorescent protein biosensors. **A–C**. The mode of action of three redox biosensors as well as their fluorescence spectra are shown as examples. **A** rxYFP is an intensiometric biosensor with a single excitation and emission peak. It was generated by introducing two cysteines in β-strands 7 and 11 (N149C, S202C) of YFP. rxYFP fluorescence decreases when a disulfide bridge forms between the two cysteines under oxidizing conditions. **B** roGFP2 is a ratiometric probe that was generated similarly as rxYFP. While the reduced form of roGFP2 has a single excitation peak at 488 nm, the oxidized form has two, at 405 and 488 nm. Ratio measurement provides a readout of the parameter of interest. **C** ECFP-RL7-EYFP is a ratiometric FRET sensor where ECFP and EYFP have been coupled by a small linker carrying 4 cysteines. Only ECFP is excited at 434 nm. Under reducing conditions, only ECFP emits fluorescence at 477 nm. Under oxidizing conditions, two disulfide bridges form in the linker, bringing both FPs in close proximity and allowing energy transfer from ECFP to EYFP, thus shifting the emission peak to 527 nm. **D** A non-exhaustive list of FP biosensors according to the physiological parameter they measure. The FPs constituting these sensors and the sensor type are indicated. Additional FP biosensors for small molecules and protein activity can be found at https://www.addgene.org/fluorescent-proteins/biosensors/. ^a^ already expressed in *Plasmodium*; ^b^ YFP-based sensors are especially sensitive to pH and must be used in parallel with a pH control probe sharing similar pKa and properties when employed to measure parameters other than pH

Beside visualization of cells, organelles and proteins, genetically encoded FPs can also be turned into versatile biosensors to measure a wide variety of physiological parameters, detect small molecules or protein/protein interactions in intact cells and specific cellular compartments [54] (Fig. 2D). This provides a key improvement compared to disruptive biochemical methods and to in vivo staining methods using fluorescent probes that cannot achieve subcellular resolution. FP sensors are divided into intensity-based and ratiometric biosensors, each having its advantages and pitfalls. In intensiometric FPs (Fig. 2A), the fluorescence intensity of a single peak increases or decreases in response to a given stimulus. Hence, these probes are sensitive to their own concentration and highly dependent on the imaging conditions (cell thickness, illumination, photobleaching), and are therefore more suited to measure changes rather than absolute parameters. On the other hand, their monochromy is an advantage for multiplexing. In ratiometric FPs (Fig. 2B), two excitation or emission peaks react differently to the same stimulus and thus information is inferred from the intensity ratio rather than intensity alone. In this group we find both dual-excitation/emission FPs and FRET-based systems (Fig. 2C). FRET, standing for Förster resonance energy transfer, is a mechanism by which energy is transferred between two fluorophores in a proximity-dependent manner that can be achieved either through rearrangement of the sensor carrying both FPs in the presence of a specific stimulus, or through the close interaction of two bait proteins, each fused to one FP, thus bringing both FPs in close proximity. Although the change of fluorescence intensity tends to be smaller for FRET sensors than for single FP sensors, FRET sensors are easier to develop and thus cover a larger range of stimuli. Of note, intensiometric sensors can be turned into ratiometric probes when fused to other FPs that are insensitive to the same stimulus. As compared to intensiometric sensors, ratiometric ones are easier to calibrate and more robust since photobleaching and probe concentration do not affect the measurement. However, these sensors require a combination of two excitation/emission filters and often present relatively small dynamic ranges.

Beside choosing the probe colour and between intensiometric and ratiometric probes for a given stimulus, three additional criteria need to be taken into account to select the most suitable sensor: (1) its sensitivity to the stimulus and (2) to other unrelated physiological parameters, and (3) its dynamic range. For instance, Peredox probes, measuring NAD + /NADH, are highly sensitive to NADH and thus, cannot be used in the mitochondrial matrix where the NADH concentration is high, hindering detection of small changes. Similarly, redox sensitive probes will only be able to measure redox potentials and detect changes if the redox potential of the cellular compartment where they are expressed is in the range of potentials that can be surveyed by the probes. As an example, a probe that is sensitive to the ratio of the reduced to oxidized forms of glutathione which maintains the redox state of the thiol groups in a cell, is fully oxidized in the endoplasmic reticulum lumen, and thus cannot be used there, while it is fully reduced in the cytoplasm and mitochondria where glutathione redox potentials are close to the lower limit of roGFP2, thus enabling detection of oxidation events. In addition, some probes may be sensitive to other unrelated parameters that can affect the reading. This is for instance the case for all YFP-based probes that are highly sensitive to pH changes. Consequently, biosensors like HyPers and Sonar that are responsive to H_2_O_2_ and NAD + /NADH, respectively, are used in parallel with SypHer that has a similar pKa but is unresponsive to H_2_O_2_ and NAD^ +^ /NADH, and can thus serve as a pH control. The fluorescence intensity of the probe and pH control should be calibrated to define a pH range where they behave similarly [55]. Ideally, a control probe based on the same FP as the sensor but insensitive to the studied stimulus should be used as reference for all unrelated environmental effects. Importantly, a weak expression, a low signal-to-noise ratio or a small dynamic range would also limit the use of the biosensor, especially in vivo, where autofluorescence is high (e.g. in vertebrate liver, mosquito tissues and iRBCs). Thus, these parameters must be evaluated in the transfected parasites to ensure the sensor can be used. In addition, stable integration of the biosensor in the parasite genome should be preferred as it overcomes several issues linked to episomal expression, including a variable/low proportion of parasites expressing the probe, variable probe expression levels between cells and a decreasing expression over time [55], but also potential interference of the selection drug with the studied parameters or with the probe itself [56, 57]. Indeed, should physiological changes be measured upon drug treatment, the absence of a direct effect of the drug on the biosensor must first be ascertained.

## Discoveries in *Plasmodium* using biosensors

Biosensors have proven useful to measure different physiological parameters in *P. falciparum* and to characterize the effect of anti-malarials. Using intensiometric EGFP and a quantitative flow cytometry assay, ratiometric pHluorin and/or SypHer, the pH of the digestive vacuole was estimated to be between 5 and 5.5, while that of the cytoplasm, the apicoplast and inside the mitochondrion is close to neutral [55, 57–59]. Of note, the accumulation of chloroquine into the digestive vacuole depends on the protonation of the haem-binding anti-malarial drug within the food vacuole [60] which led to the hypothesis that changes in the pH of the organelle could contribute to the resistance phenotype of some strains. However, neither EGFP [59] nor pHluorin [58] detected differences in the pH of the digestive vacuole between resistant and sensitive strains. Interestingly, haem quantification using the CH_49_Y FRET sensor demonstrated that the amount of free haem remains relatively low and tightly regulated in the parasite cytoplasm, even during extensive haemoglobin digestion at the trophozoite stage, but increases upon chloroquine treatment, likely generating oxidative stress [61]. Both hGrx1-roGFP2 and sfroGFP2, presenting a brighter signal and thus more adapted to measure redox variations across asexual stages, including merozoites, have been used to measure the redox potential of glutathione in the cytoplasm (− 300 mV), the apicoplast (− 330 mV) and mitochondria (− 270 mV) [56, 62, 63]. They are suitable to detect nitrosative and oxidative stress in the parasite [62] and can be used for single cell imaging and plate reader assays [56]. Notably, most anti-malarial drugs do not interact directly with hGrx1-roGFP2 and sfroGFP2 [56, 57, 62], underlying their utility to explore drug-induced changes in the redox milieu. Multiple drugs have a pro-oxidant effect on the cytosolic pool of glutathione, including quinolones and artemisinin [56, 62], while the mitochondrial glutathione pool remains unaffected upon treatment with these drugs [57], indicating both pools are relatively independent. The pro-oxidant effect is further supported by an increase in H_2_O_2_ levels detected by a roGFP2-Orp1 probe in parasites treated with these drugs [55]. Using the Yellow Cameleon Nano FRET sensor (YC-nano), Ca^2+^ levels were shown to be low in trophozoites (30 nM), but high in other stages (> 300 mM), and exposure to artemisinin did not lead to any changes in Ca^2+^ concentrations in the cytosol. However, these experiments were performed using the Yellow Cameleon Nano FRET sensor, hence subtle changes in the Ca^2+^ concentration might not have been detected [64]. Of note, GcaMP3 was used to evaluate the dynamics of the parasites Ca^2+^ stores [65]. Finally, FRET has also recently been used to follow interactions between the nutrient channel proteins CLAG3 and RopH2 that were fused to two GFP derivatives, mCerulan and mVenus, respectively [66]. Although not yet applied in *Plasmodium*, split-FPs and Bioluminescence Resonance Energy Transfer (BRET) might provide alternatives to FRET to monitor proximity in live cells while increasing the fluorescence signal or limiting autofluorescence, respectively. Indeed, in BRET, the energy is transferred from a donor luciferase to an acceptor FP and thus, no excitation light is required. It is worth mentioning that the effect of BRET can also be undesirable if FP and luciferase are fused but fluorescence and bioluminescence have different uses, e.g. for FP based cell sorting by flow cytometry and luciferase based luminometric measurement of the parasite burden in vivo. In this case the bioluminescence might be quenched, possibly reducing intensity in luminometric measurements. To avoid BRET in such experimental settings, it is important to consider that the wavelength of the emitted bioluminescence does not overlap with the excitation wavelength of the fused FP.

## FPs beyond GFP and DsRed

With the continuous discovery of new FPs and improvement of existing FP scaffolds, the available collection of genetically encoded fluorescent reporters has reached a dimension that is difficult to survey. So far (March 2024), 1058 proteins are listed in the FP database that is probably not exhaustive [67]. This wide variety of available FPs, including many method-specific fluorescent reporters, can make choosing an optimal FP difficult. In addition, the genetic modification of *Plasmodium* requires significantly more time and effort than the transfection of e.g. mammalian cells, which likely explains why studies on *Plasmodium* still often rely on variants of GFP and dsRed that have proven reliable over many years. These prototypic FPs and their derivatives, mEmerald, mVenus, mTurquoise and mCherry, perform well for most applications [68–71]. Nevertheless, FP scaffolds that outperform GFP or DsRed have been discovered or engineered. A recent example of a red FP with significantly improved quantum yield and brightness compared to mCherry is mScarlet [72]. mScarlet was developed based on an in silico scaffold that combined sequences from different known RFPs, followed by several rounds of mutagenesis-based optimization. By starting mutagenesis with an artificially designed scaffold, the structure could be adapted to eliminate areas favouring dimerization, making mScarlet a true monomeric FP [72]. Due to its excellent properties, mScarlet has already been applied as a reporter for protein export in *P. falciparum* infected red blood cells [73] and as a marker for successful transgenesis in *P. berghei* [74]. mNeonGreen (mNG), emitting light in the green/yellow spectrum, is three times brighter than EGFP and matures significantly faster while similar in terms of size and quantum yield, making it therefore an excellent alternative to GFPs and YFPs. Of note, mNG has already been used in *P. berghei* for C-terminal tagging of the myosin A tail domain interacting protein (MTIP) and α-tubulin II [50, 75], and as a stage-specific fluorescent reporter [50]. While originating from different scaffolds, mScarlet, mNG as well as GFP, dsRed and their derivatives have in common that they possess an intrinsically encoded chromophore. This is not always the case as many FPs rely on cofactors to be functional. Since the cofactor forms the chromophore, many cofactor-dependent FPs have significantly smaller sizes (12–18 kDa) compared to prototypic FPs (~ 25 kDa) [76]. Biliverdin-dependent FPs constitute a large family [77–82], most of them with an emission and excitation maximum in the near-infrared (NIR-FPs) which is not reached by prototypical FPs. Therefore, NIR FPs complement the existing colour palette of BFPs, GFPs, YFPs and RFPs. A promising NIR FP is the recently described miRFP670nano, which is with a molecular weight of 17.1 kDa, the smallest NIR-FP known to date and even allows inter-domain protein tagging [82]. The only known bilirubin-dependent FP, UnaG, was discovered in the Japanese eel “Unagi”, [83]. UnaG combines a small size (15.6 kDa) with excitation/emission properties similar to YFPs while its chromophore maturation does not require oxygen. Obstacles in the use of miRFP670nano and UnaG rely in their dependency on their cofactors biliverdin and bilirubin, respectively. The level of both cofactors inside *Plasmodium* infected red blood cells is low [84] which suggests they may need cofactor supplementation when expressed in blood stages [85]. Therefore, the use of UnaG and miRFP670nano as well as FPs with similar properties will likely remain restricted to niche applications in *Plasmodium,* like deep tissue imaging of sporozoites and liver stages (NIR-FPs) and interdomain tagging approaches to study protein processing. In contrast, mNG and mScarlet, that can be used in a similar way to GFP and DsRed variants, have a much broader range of applications. mNG differs from GFP only in its emission spectrum, which is shifted to yellow, thus changing the overlap with other fluorophores. One difference of mScarlet from other RFPs is its long maturation time, which probably makes it unsuitable for the detection of rapid transcriptional changes in gene expression as observed in stage transitions during *Plasmodium* development.

## Conclusions

While genetically encoded fluorescent reporters have become an indispensable tool for research in *Plasmodium* most work is still based on a few classical FPs*.* The historical difficulties in generating transgenic *P. falciparum* have restricted the use of FPs and the development of genetic tools which may explain the lack of strong promoters and intergenic integration sites to expand the toolbox of fluorescent *P. falciparum* reporter lines. The use of *Plasmodium* artificial chromosomes (see "List of abbreviations"), that are centromere-containing plasmids stably inherited even in the absence of drug pressure [86], could potentially provide and elegant solution for the stable expression of ectopic transgenes without altering the parasite genome. Nevertheless, they have hardly been used so far and have yet to prove their usefulness. Two major breakthroughs in the field are the adaptation of the CRISPR/Cas9 system to *Plasmodium* and the development of a selection linked integration methodology (see "List of abbreviations") which have opened up a new repertory of possible genetic modifications, notably in *P. falciparum* [87, 88]. Therefore, it can be expected to see a dramatic increase in sophisticated experimental genetic tools in malariology within the next years. This review aims to offer valuable insights into the application of FPs in *Plasmodium* and to encourage the updating of the community collection of plasmids to incorporate the latest generation of FPs.

## List of abbreviations

*CRISPR/Cas9 System*: Nuclease (Cas9)-mediated bacterial anti-phage defense system called Clustered Regularly Interspaced Palindromic Repeats (CRISPR) which can be exploited to mediate genetic changes by providing artificial guide RNAs defining the cutting site for the Cas9 nuclease, notably in a double-stranded DNA genome

*Dihydrofolate reductase and dihydrofolate reductase-thymidylate synthase (DHFR/DHFR-TS)*: The dihydrofolate reductase and the bifunctional enzyme dihydrofolate reductase-thymidylate synthase convert dihydrofolate into tetrahydrofolate which serves as a cofactor in the synthesis of purines and certain amino acids; the essentiality of tetrahydrofolate makes *Plasmodium* dihydrofolate reductase an ideal target for drug selection using pyrimethamine (see below) or WR99210.

*Episome*: Extra-chromosomal genetic material which replicates autonomously in the parasite. Episomes can be plasmids although the mechanism by which *Plasmodium* maintains and replicates bacterial DNA remains unknown. Selection of transfected parasites carrying episomes requires a selectable marker, and episomes are only retained upon drug pressure. The number of episomes per parasite varies extensively.

*Plasmodium artificial chromosomes (PACs)*: are based on a plasmid named pFCEN containing an artificial *P. falciparum* centromere, that allows transfected parasites to retain the plasmid as a single copy (like a chromosome), even in the absence of drug pressure, which is not the case for episomes (see above).

*Pyrimethamine*: a drug which competes with dihydrofolate at the active site of dihydrofolate reductases and, as a consequence, inhibits its enzymatic activity; human dihydrofolate reductase as well as mutated variants of protozoan dihydrofolate reductases, and dihydrofolate reductase-thymidylate synthases are resistant to inhibition and can be used as positive selection markers during transgenesis.

*Selection-linked integration (SLI)*: A method to rapidly select for parasite transformants in *P. falciparum* using plasmids that contain two positive selection markers. The first marker selects for uptake and episomal inheritance of transfected DNA, while the second marker is only expressed upon genomic integration. The formation of the second resistance is achieved by fusing the selection marker with the open reading frame of the protein of interest via a self-cutting peptide (e.g. T2A). The enrichment of episome-containing parasites significantly increases the probability of finding parasites that have integrated the plasmid into the genome when selecting for the second selection marker in a time-delayed manner

## Data Availability

Not applicable.

## References

[1] De Niz M, Burda PC, Kaiser G, Del Portillo HA, Spielmann T, Frischknecht F (2017). Progress in imaging methods: insights gained into *Plasmodium* biology. Nat Rev Microbiol.

[2] Birnbaum J, Scharf S, Schmidt S, Jonscher E, Maria Hoeijmakers WA, Flemming S (2020). A Kelch13-defined endocytosis pathway mediates artemisinin resistance in malaria parasites. Science.

[3] Filarsky M, Fraschka SA, Niederwieser I, Brancucci NMB, Carrington E, Carrió E (2018). GDV1 induces sexual commitment of malaria parasites by antagonizing HP1-dependent gene silencing. Science.

[4] Zeeshan M, Shilliday F, Liu T, Abel S, Mourier T, Ferguson DJP (2019). *Plasmodium* kinesin-8X associates with mitotic spindles and is essential for oocyst development during parasite proliferation and transmission. PLoS Pathog.

[5] Singer M, Frischknecht F (2021). Fluorescent tagging of *Plasmodium* circumsporozoite protein allows imaging of sporozoite formation but blocks egress from oocysts. Cell Microbiol.

[6] Ripp J, Kehrer J, Smyrnakou X, Tisch N, Tavares J, Amino R (2021). Malaria parasites differentially sense environmental elasticity during transmission. EMBO Mol Med.

[7] Hopp CS, Kanatani S, Archer NK, Miller RJ, Liu H, Chiou KK (2021). Comparative intravital imaging of human and rodent malaria sporozoites reveals the skin is not a species-specific barrier. EMBO Mol Med.

[8] Sturm A, Amino R, van de Sand C, Regen T, Retzlaff S, Rennenberg A (2006). Manipulation of host hepatocytes by the malaria parasite for delivery into liver sinusoids. Science.

[9] Burda P-C, Roelli MA, Schaffner M, Khan SM, Janse CJ, Heussler VT (2015). A *Plasmodium* phospholipase is involved in disruption of the liver stage parasitophorous vacuole membrane. PLOS Pathog.

[10] Janse CJ, Franke-Fayard B, Mair GR, Ramesar J, Thiel C, Engelmann S (2006). High efficiency transfection of *Plasmodium berghei* facilitates novel selection procedures. Mol Biochem Parasitol.

[11] Siciliano G, Alano P (2015). Enlightening the malaria parasite life cycle: bioluminescent *Plasmodium* in fundamental and applied research. Front Microbiol.

[12] Miyazaki Y, Vos MW, Geurten FJA, Bigeard P, Kroeze H, Yoshioka S (2023). A versatile *Plasmodium falciparum* reporter line expressing NanoLuc enables highly sensitive multi-stage drug assays. Commun Biol.

[13] De Niz M, Stanway RR, Wacker R, Keller D, Heussler VT (2016). An ultrasensitive NanoLuc-based luminescence system for monitoring *Plasmodium berghei* throughout its life cycle. Malar J.

[14] Ponzi M, Siden-Kiamos I, Bertuccini L, Curra C, Kroeze H, Camarda G (2009). Egress of *Plasmodium berghei* gametes from their host erythrocyte is mediated by the MDV-1/PEG3 protein. Cell Microbiol.

[15] Matz JM, Goosmann C, Matuschewski K, Kooij TWA (2018). An unusual prohibitin regulates malaria parasite mitochondrial membrane potential. Cell Rep.

[16] Rathnapala UL, Goodman CD, McFadden GI (2017). A novel genetic technique in *Plasmodium berghei* allows liver stage analysis of genes required for mosquito stage development and demonstrates that de novo heme synthesis is essential for liver stage development in the malaria parasite. PLoS Pathog.

[17] Gehde N, Hinrichs C, Montilla I, Charpian S, Lingelbach K, Przyborski JM (2009). Protein unfolding is an essential requirement for transport across the parasitophorous vacuolar membrane of *Plasmodium falciparum*. Mol Microbiol.

[18] Boucher MJ, Yeh E (2019). Disruption of apicoplast biogenesis by chemical stabilization of an imported protein evades the delayed-death phenotype in malaria parasites. mSphere.

[19] Van Dijk MR, Waters AP, Janse CJ (1995). Stable transfection of malaria parasite blood stages. Science.

[20] Van Spaendonk RML, Ramesar J, Van Wigcheren A, Eling W, Beetsma AL, Van Gemert GJ (2001). Functional equivalence of structurally distinct ribosomes in the malaria parasite. Plasmodium Berghei J Biol Chem.

[21] Franke-Fayard B, Trueman H, Ramesar J, Mendoza J, Van Der Keur M, Van Der Linden R (2004). A *Plasmodium berghei* reference line that constitutively expresses GFP at a high level throughout the complete life cycle. Mol Biochem Parasitol.

[22] Helm S, Lehmann C, Nagel A, Stanway RR, Horstmann S, Llinas M (2010). Identification and characterization of a liver stage-specific promoter region of the malaria parasite *Plasmodium*. PLoS ONE.

[23] van Dijk MR, van Schaijk BCL, Khan SM, van Dooren MW, Ramesar J, Kaczanowski S (2010). Three members of the 6-cys protein family of *Plasmodium* play a role in gamete fertility. PLoS Pathog.

[24] van Schaijk BCL, van Dijk MR, van de Vegte-Bolmer M, van Gemert GJ, van Dooren MW, Eksi S (2006). Pfs47, paralog of the male fertility factor Pfs48/45, is a female specific surface protein in *Plasmodium falciparum*. Mol Biochem Parasitol.

[25] Miyazaki S, Yang ASP, Geurten FJA, Marin-Mogollon C, Miyazaki Y, Imai T (2020). Generation of novel *Plasmodium falciparum* NF135 and NF54 lines expressing fluorescent reporter proteins under the control of strong and constitutive promoters. Front Cell Infect Microbiol.

[26] Portugaliza HP, Llorà-Batlle O, Rosanas-Urgell A, Cortés A (2019). Reporter lines based on the gexp02 promoter enable early quantification of sexual conversion rates in the malaria parasite *Plasmodium falciparum*. Sci Rep.

[27] Ishino T, Boisson B, Orito BB, Lacroix C, Bischoff E, Loussert C (2009). LISP1 is important for the egress of *Plasmodium berghei* parasites from liver cellsc. Cell Microbiol.

[28] Hoshizaki J, Jagoe H, Lee MCS (2022). Efficient generation of mNeonGreen *Plasmodium falciparum* reporter lines enables quantitative fitness analysis. Front Cell Infect Microbiol.

[29] Suárez-Cortés P, Costa G, Andres M, Eyermann D, Kreschel C, Spohr L (2023). Generation of a *Plasmodium falciparum* reporter line for studies of parasite biology throughout the life cycle. bioRxiv.

[30] Th M, Tony T, Jenny T, Mohammed S, Ruth F, E. Wickham ME,  (2001). *Plasmodium falciparum* homologue of the genes for *Plasmodium vivax* and *Plasmodium yoelii* adhesive proteins, which is transcribed but not translated. Infect Immun.

[31] Collier S, Pietsch E, Dans M, Ling D, Tavella TA, Lopaticki S (2023). *Plasmodium falciparum* formins are essential for invasion and sexual stage development. Commun Biol.

[32] Matz JM, Kooij TWA (2015). Towards genome-wide experimental Genetics in the in vivo malaria model parasite *Plasmodium berghei*. Pathog Glob Health.

[33] Deligianni E, Morgan RN, Bertuccini L, Kooij TWA, Laforge A, Nahar C (2011). Critical role for a stage-specific actin in male exflagellation of the malaria parasite. Cell Microbiol.

[34] Adjalley SH, Johnston GL, Li T, Eastman RT, Ekland EH, Eappen AG (2011). Quantitative assessment of *Plasmodium falciparum* sexual development reveals potent transmissionblocking activity by methylene blue. Proc Natl Acad Sci USA.

[35] Klug D, Mair GR, Frischknecht F, Douglas RG (2016). A small mitochondrial protein present in myzozoans is essential for malaria transmission. Open Biol.

[36] Janse CJ, Franke-Fayard B, Waters AP (2006). Selection by flow-sorting of genetically transformed, GFP-expressing blood stages of the rodent malaria parasite. Plasmodium Berghei Nat Protoc.

[37] Manzoni G, Briquet S, Risco-Castillo V, Gaultier C, Topçu S, Ivănescu ML (2015). A rapid and robust selection procedure for generating drug-selectable marker-free recombinant malaria parasites. Sci Rep.

[38] Bane K, Lepper S, Kehrer J, Sattler JM, Singer M, Reinig M (2016). The actin filament-binding protein coronin regulates motility in *Plasmodium* sporozoites. PLoS Pathog.

[39] Talman AM, Blagborough AM, Sinden RE (2010). A *Plasmodium falciparum* strain expressing GFP throughout the parasite’s life-cycle. PLoS ONE.

[40] Vaughan AM, Mikolajczak SA, Camargo N, Lakshmanan V, Kennedy M, Lindner SE (2012). A transgenic *Plasmodium falciparum* NF54 strain that expresses GFP-luciferase throughout the parasite life cycle. Mol Biochem Parasitol.

[41] McLean KJ, Straimer J, Hopp CS, Vega-Rodriguez J, Small-Saunders JL, Kanatani S (2019). Generation of transmission-competent human malaria parasites with chromosomally-integrated fluorescent reporters. Sci Rep.

[42] Kooij TWA, Rauch MM, Matuschewski K (2012). Expansion of experimental genetics approaches for *Plasmodium berghei* with versatile transfection vectors. Mol Biochem Parasitol.

[43] Matz JM, Matuschewski K, Kooij TWA (2013). Two putative protein export regulators promote *Plasmodium* blood stage development in vivo. Mol Biochem Parasitol.

[44] Manzoni G, Briquet S, Risco-Castillo V, Gaultier C, Topçu S, Ivǎnescu ML (2014). A rapid and robust selection procedure for generating drug-selectable marker-free recombinant malaria parasites. Sci Rep.

[45] Vos MW, Stone WJR, Koolen KM, Van Gemert GJ, Van Schaijk B, Leroy D (2015). A semi-automated luminescence based standard membrane feeding assay identifies novel small molecules that inhibit transmission of malaria parasites by mosquitoes. Sci Rep.

[46] Mogollon CM, Van Pul FJA, Imai T, Ramesar J, Chevalley-Maurel S, De Roo GM (2016). Rapid generation of marker-free P. falciparum fluorescent reporter lines using modified CRISPR/Cas9 constructs and selection protocol. PLoS ONE.

[47] Marin-Mogollon C, Salman AM, Koolen KMJ, Bolscher JM, Van Pul FJA, Miyazaki S (2019). A *P. falciparum* NF54 reporter line expressing mCherry-luciferase in gametocytes, sporozoites, and liver-stages. Front Cell Infect Microbiol..

[48] Spaccapelo R, Janse CJ, Caterbi S, Franke-Fayard B, Bonilla JA, Syphard LM (2010). Plasmepsin 4-deficient *Plasmodium berghei* are virulence attenuated and induce protective immunity against experimental malaria. Am J Pathol.

[49] Franke-Fayard B, Janse CJ, Cunha-rodrigues M, Ramesar J, Büscher P, Que I (2005). Murine malaria parasite sequestration: CD36 is the major receptor, but cerebral pathology is unlinked. PNAS.

[50] Mori T, Hirai M, Mita T (2019). See-through observation of malaria parasite behaviors in the mosquito vector. Sci Rep.

[51] Hopp CS, Chiou K, Ragheb DRT, Salman AM, Khan SM, Liu AJ (2015). Longitudinal analysis of *Plasmodium* sporozoite motility in the dermis reveals component of blood vessel recognition. Elife.

[52] Natarajan R, Thathy V, Mota MM, Hafalla JCR, Ménard R, Vernick KD (2001). Fluorescent *Plasmodium berghei* sporozoites and pre-erythrocytic stages: a new tool to study mosquito and mammalian host interactions with malaria parasites. Cell Microbiol.

[53] Khan SM, Kroeze H, Franke-Fayard B, Janse CJ (2013). Standardization in generating and reporting genetically modified rodent malaria parasites: the RMgmDB database. Methods Mol Biol.

[54] Bilan DS, Belousov VV (2017). New tools for redox biology: from imaging to manipulation. Free Radical Biol Med.

[55] Rahbari M, Rahlfs S, Jortzik E, Bogeski I, Becker K (2017). H_2_O_2_ dynamics in the malaria parasite *Plasmodium falciparum*. PLoS ONE.

[56] Schuh AK, Rahbari M, Heimsch KC, Mohring F, Gabryszewski SJ, Weder S (2018). Stable integration and comparison of hGrx1-roGFP2 and sfroGFP2 redox probes in the malaria parasite *Plasmodium falciparum*. ACS Infect Dis.

[57] Mohring F, Rahbari M, Zechmann B, Rahlfs S, Przyborski JM, Meyer AJ (2017). Determination of glutathione redox potential and pH value in subcellular compartments of malaria parasites. Free Radic Biol Med.

[58] Kuhn Y, Rohrbach P, Lanzer M (2007). Quantitative pH measurements in P*lasmodium falciparum*-infected erythrocytes using pHluorin. Cell Microbiol.

[59] Klonis N, Tan O, Jackson K, Goldberg D, Klemba M, Tilley L (2007). Evaluation of pH during cytostomal endocytosis and vacuolar catabolism of haemoglobin in *Plasmodium falciparum*. Biochem J.

[60] Homewood CA, Warhurst DC, Peters W, Baggaley VC (1972). Lysosomes, pH and the anti-malarial action of chloroquine. Nature.

[61] Abshire JR, Rowlands CJ, Ganesan SM, So PTC, Niles JC (2017). Quantification of labile heme in live malaria parasites using a genetically encoded biosensor. Proc Natl Acad Sci U S A.

[62] Kasozi D, Mohring F, Rahlfs S, Meyer AJ, Becker K (2013). Real-Time Imaging of the intracellular glutathione redox potential in the malaria parasite *Plasmodium falciparum*. PLoS Pathog.

[63] Mohring F, Jortzik E, Becker K (2016). Comparison of methods probing the intracellular redox milieu in *Plasmodium falciparum*. Mol Biochem Parasitol.

[64] Pandey K, Ferreira PE, Ishikawa T, Nagai T, Kaneko O, Yahata K (2016). Ca^2+^ monitoring in *Plasmodium falciparum* using the yellow cameleon-Nano biosensor. Sci Rep.

[65] Pereira LB, Thomas SJ, Silva ALA, Bartlett PJ, Thomas AP, Garcia CRS (2020). The genetic Ca^2+^ sensor GCaMP3 reveals multiple Ca^2+^ stores differentially coupled to Ca2^+^ entry in the human malaria parasite *Plasmodium falciparum*. J Biol Chem.

[66] Ahmad M, Manzella-Lapeira J, Saggu G, Ito D, Brzostowski JA, Desai SA (2020). Live-cell fret reveals that malaria nutrient channel proteins clag3 and rhoph2 remain associated throughout their tortuous trafficking. MBio.

[67] Lambert TJ (2019). FPbase: a community-editable fluorescent protein database. Nat Methods.

[68] Shaner NC, Campbell RE, Steinbach PA, Giepmans BNG, Palmer AE, Tsien RY (2004). Improved monomeric red, orange and yellow fluorescent proteins derived from Discosoma sp. red fluorescent protein. Nat Biotechnol.

[69] Kremers GJ, Goedhart J, Van Munster EB, Gadella TWJ (2006). Cyan and yellow super fluorescent proteins with improved brightness, protein folding, and FRET förster radius. Biochemistry.

[70] Goedhart J, Van Weeren L, Hink MA, Vischer NOE, Jalink K, Gadella TWJ (2010). Bright cyan fluorescent protein variants identified by fluorescence lifetime screening. Nat Methods.

[71] Balleza E, Kim JM, Cluzel P (2018). Systematic characterization of maturation time of fluorescent proteins in living cells. Nat Methods.

[72] Bindels DS, Haarbosch L, van Weeren L, Postma M, Wiese KE, Mastop M (2017). mScarlet: a bright monomeric red fluorescent protein for cellular imaging. Nat Methods.

[73] Mesén-Ramírez P, Bergmann B, Tran TT, Garten M, Stäcker J, Naranjo-Prado I (2019). EXP1 is critical for nutrient uptake across the parasitophorous vacuole membrane of malaria parasites. PLoS Biol.

[74] Jiang Y, Wei J, Cui H, Liu C, Zhi Y, Jiang ZZ (2020). An intracellular membrane protein GEP1 regulates xanthurenic acid induced gametogenesis of malaria parasites. Nat Commun.

[75] Shinzawa N, Nishi T, Hiyoshi F, Motooka D, Yuda M, Iwanaga S (2020). Improvement of CRISPR/Cas9 system by transfecting Cas9-expressing *Plasmodium berghei* with linear donor template. Commun Biol.

[76] Rodriguez EA, Campbell RE, Lin JY, Lin MZ, Miyawaki A, Palmer AE (2017). The growing and glowing toolbox of fluorescent and photoactive proteins. Trends Biochem Sci.

[77] Shcherbakova DM, Cammer NC, Huisman TM, Vladislav V, Hodgson L, Biology S (2018). Direct multiplex imaging and optogenetics of RhoGTPases enabled by near-infrared FRET. Nat Chem Biol.

[78] Yu D, Baird MA, Allen JR, Howe ES, Klassen MP, Reade A (2015). A naturally-monomeric infrared fluorescent protein for protein labeling in vivo. Nat Methods.

[79] Ghosh S, Yu CL, Ferraro DJ, Sudha S, Pal SK, Schaefer WF (2016). Blue protein with red fluorescence. Proc Natl Acad Sci U S A.

[80] Rodriguez EA, Tran GN, Gross LA, Crisp JL, Shu X, Lin JY (2016). A far-red fluorescent protein evolved from a cyanobacterial phycobiliprotein. Nat Methods.

[81] Ding WL, Hou YN, Tan ZZ, Jiang SP, Miao D, Losi A (2018). Far-red acclimating cyanobacterium as versatile source for bright fluorescent biomarkers. Biochim Biophys Acta Mol Cell Res.

[82] Oliinyk OS, Shemetov AA, Pletnev S, Shcherbakova DM, Verkhusha VV (2019). Smallest near-infrared fluorescent protein evolved from cyanobacteriochrome as versatile tag for spectral multiplexing. Nat Commun.

[83] Kumagai A, Ando R, Miyatake H, Greimel P, Kobayashi T, Hirabayashi Y (2013). A bilirubin-inducible fluorescent protein from eel muscle. Cell.

[84] Sigala PA, Crowley JR, Hsieh S, Henderson JP, Goldberg DE (2012). Direct tests of enzymatic heme degradation by the malaria parasite *Plasmodium falciparum*. J Biol Chem.

[85] Qian Y, Piatkevich KD, Mc Larney B, Abdelfattah AS, Mehta S, Murdock MH (2019). A genetically encoded near-infrared fluorescent calcium ion indicator. Nat Methods.

[86] Iwanaga S, Kato T, Kaneko I, Yuda M (2012). Centromere plasmid: a new genetic tool for the study of *Plasmodium falciparum*. PLoS ONE.

[87] Ghorbal M, Gorman M, MacPherson CR, Martins RM, Scherf A, Lopez-Rubio JJ (2014). Genome editing in the human malaria parasite *Plasmodium falciparum* using the CRISPR-Cas9 system. Nat Biotechnol.

[88] Birnbaum J, Flemming S, Reichard N, Soares AB, Mesen-Ramirez P, Jonscher E (2017). A genetic system to study Plasmodium falciparum protein function. Nat Meth.

[89] Ukegbu CV, Cho JS, Christophides GK, Vlachou D (2015). Transcriptional silencing and activation of paternal DNA during *Plasmodium berghei* zygotic development and transformation to oocyst. Cell Microbiol.

[90] Wang Z, Liu M, Liang X, Siriwat S, Li X, Chen X (2014). A flow cytometry-based quantitative drug sensitivity assay for all *Plasmodium falciparum* gametocyte stages. PLoS ONE.

[91] Baragaña B, Hallyburton I, Lee MCS, Norcross NR, Grimaldi R, Otto TD (2015). A novel multiple-stage antimalarial agent that inhibits protein synthesis. Nature.

